# Formose reaction controlled by boronic acid compounds

**DOI:** 10.3762/bjoc.12.263

**Published:** 2016-12-08

**Authors:** Toru Imai, Tomohiro Michitaka, Akihito Hashidzume

**Affiliations:** 1 Department of Macromolecular Science, Graduate School of Science, Osaka University, 1-1 Machikaneyama-cho, Toyonaka, Osaka 560-0043, Japan

**Keywords:** boronic acid compounds, formose reaction, sodium phenylboronate, sodium 4-vinylphenylboronate/sodium 4-styrenesulfonate copolymer, sugar alcohols, sugars

## Abstract

Formose reactions were carried out in the presence of low molecular weight and macromolecular boronic acid compounds, i.e., sodium phenylboronate (SPB) and a copolymer of sodium 4-vinylphenylboronate with sodium 4-styrenesulfonate (pVPB/NaSS), respectively. The boronic acid compounds provided different selectivities; sugars of a small carbon number were formed favorably in the presence of SPB, whereas sugar alcohols of a larger carbon number were formed preferably in the presence of pVPB/NaSS.

## Findings

When an aqueous solution of formaldehyde is warmed in the presence of a basic catalyst, a mixture of sugars and sugar alcohols, i.e., ‘formose’, is obtained. This reaction is called ‘formose reaction’ and was first reported by Butlerow, a Russian chemist, in 1861 [1]. Studies on formose reactions have revealed that the formose reaction consists of several elemental reaction steps, e.g., acyloin condensation, aldol reaction, retro-aldol reaction, aldose–ketose isomerization, and Cannizzaro reaction, and the product, formose, is a complicated mixture of more than thirty species of sugars and sugar alcohols including non-natural ones, i.e., branched and L-isomers [2]. The formose reaction has being considered as a possible pathway for sugar formation under prebiotic conditions [3–5]. Some pioneering works from late 1970’s to 1980’s have demonstrated that some sugars or sugar alcohols are selectively formed by optimizing the conditions of the formose reaction [6–15]. However, the selective formose reaction is still an important subject of investigation [16].

It is known that boronic acid compounds form esters with diols, e.g., sugars [17–18]. Boronic acid compounds may thus stabilize the sugars formed in the formose reaction [19]. Since the stability of boronic acid esters is dependent on the relative configuration of two hydroxy groups, i.e., the type of sugar [17–18], boronic acid compounds may provide some selectivity to formose reaction. In this study, we have thus carried out formose reactions in the presence of low molecular weight and macromolecular boronic acid compounds.

**Scheme 1:**
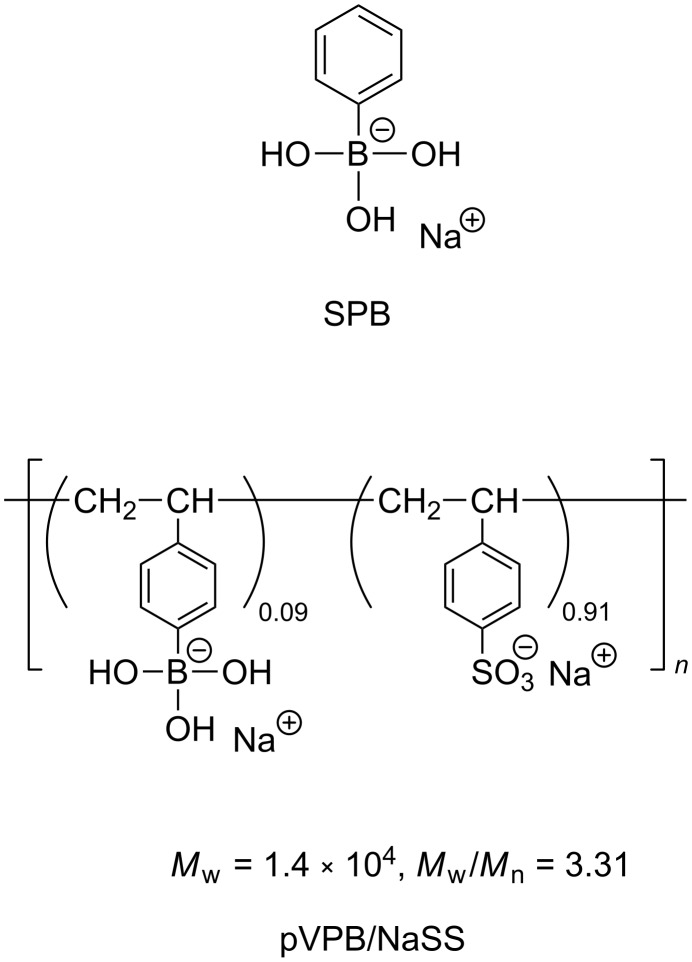
Structures of the boronic acid compounds used in this study (i.e., SPB and pVPB/NaSS).

**Figure 1:**
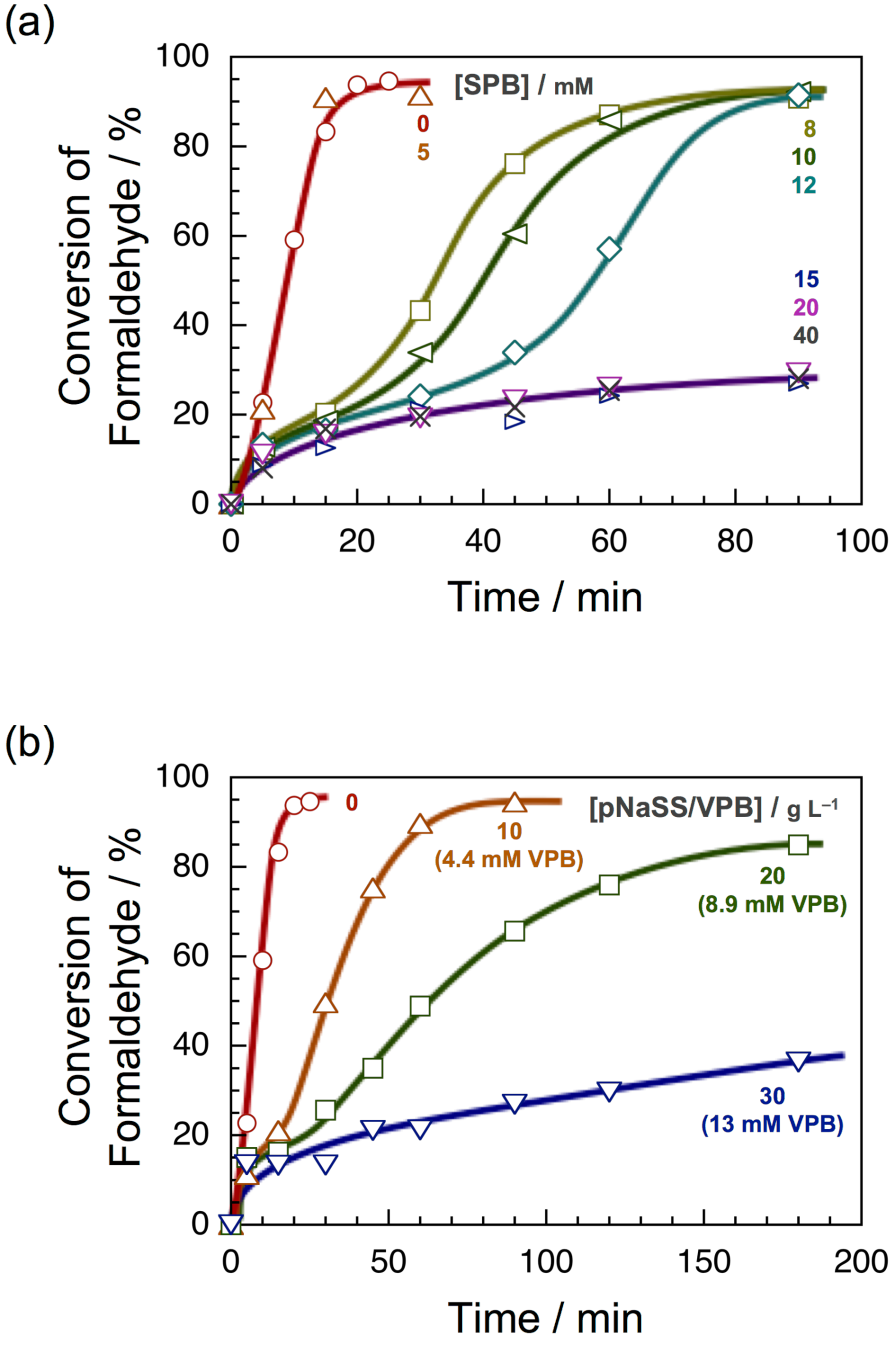
Time–conversion plots for the formose reaction using 200 mM formaldehyde, 20 mM calcium hydroxide, and 6.9 mM cocatalyst in the presence of SPB (a) and pVPB/NaSS (b); fructose and glyceraldehyde was used as a cocatalyst for SPB and pVPB/NaSS, respectively. The curves are drawn as a guide for the eye.

Sodium phenylboronate (SPB) and a copolymer of sodium 4-vinylphenylboronate with sodium 4-styrenesulfonate (pVPB/NaSS) were used as a low molecular weight boronic acid compound and a boronic acid polymer, respectively (Scheme 1). The copolymer was prepared by radical copolymerization at a molar ratio of 1:10 in monomer feed. The formose reaction was carried out using a solution containing 200 mM formaldehyde and 20 mM calcium hydroxide at 60 °C. Fructose or glyceraldehyde was employed as a cocatalyst because the formose reaction did not proceed in the presence of a boronic acid compound without cocatalyst. The conversions of formose reactions were determined by the acetylacetone method [20–21]. Figure 1a shows a typical example of the time–conversion plots for formose reactions in the presence of SPB. In the presence of 5 mM SPB, the time–conversion plots are almost the same as those for formose reactions in the absence of boronic acid compounds, indicative of no effect of SPB at a lower concentration. At 8, 10, and 12 mM, the conversion shows a small onset and then increases gradually. After ca. 30 min, the conversion starts to increase significantly and then reaches a quantitative conversion. At concentrations ≥15 mM, the conversion is ca. 30% even after 90 min. These data indicate that SPB retards the formose reaction. Figure 1b shows the time–conversion plots for formose reactions carried out in the presence of pVPB/NaSS. The formose reaction is retarded more significantly at higher concentrations. At 30 g L^−1^, the conversion was ca. 40% even after 180 min, indicating that pVPB/NaSS also retards the formose reaction. Figure S1 in Supporting Information File 1 compares the time–conversion data for SPB and pVPB/NaSS. This figure indicates that pVPB/NaSS retards the formose reaction more significantly than SPB does at similar concentrations of boronic acid residues. In a separate experiment, the formose reaction did not proceed significantly in the presence of 100 g L^−1^ poly(sodium 4-styrenesulfonate) presumably because sulfonate residues capture calcium ions. These observations indicate that the stronger retardation effect of pVPB/NaSS is ascribable to both boronic acid and sulfonate residues.

**Figure 2:**
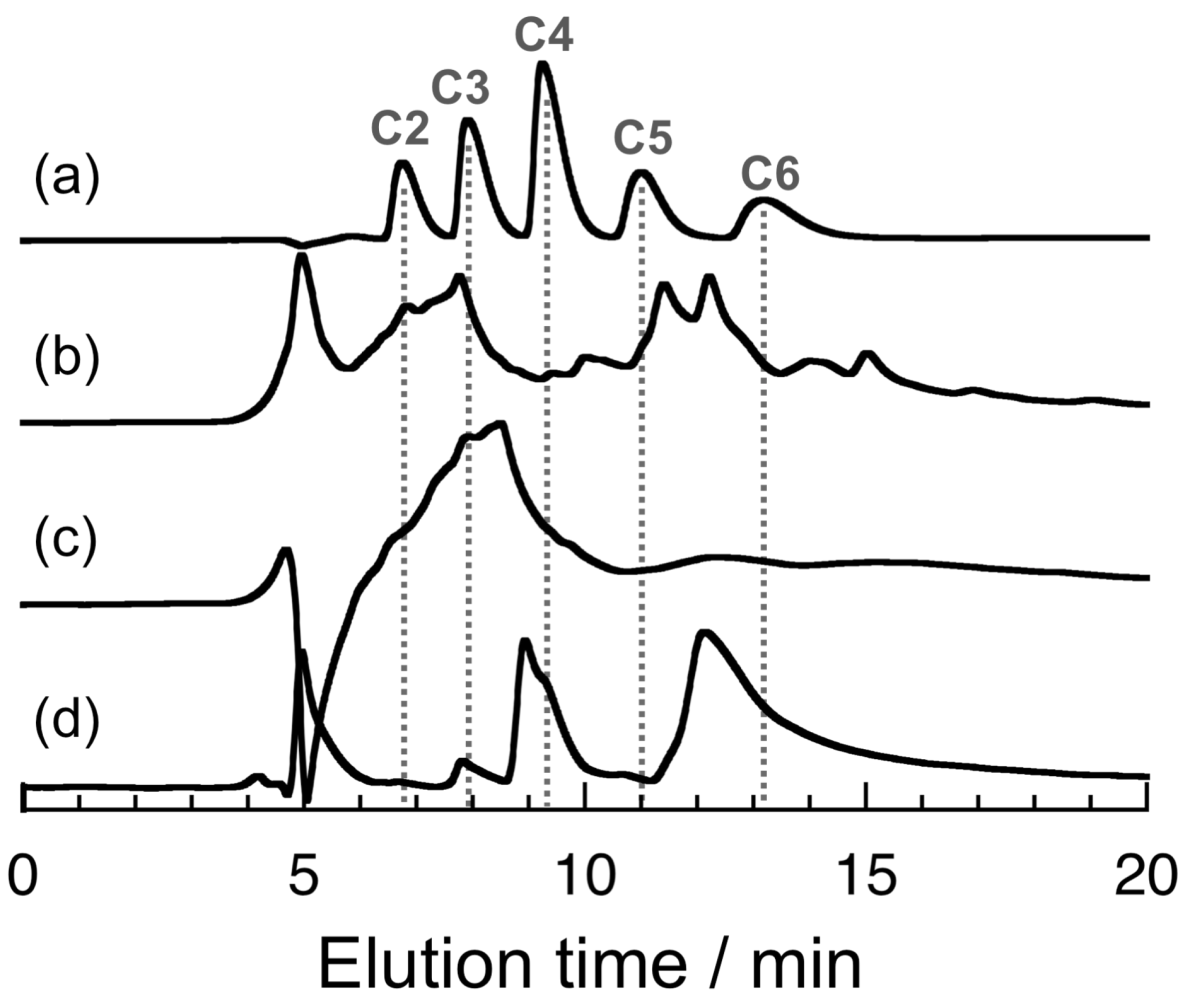
HPLC charts for standard samples (a) and products of the formose reaction in the absence (b) and presence of 10 mM SPB (c) and 20 g L^−1^ pVPB/NaSS (d); the reaction times were 20, 90, and 180 min for (b), (c), and (d), respectively.

The product was purified by dialysis against water and treatment with ion-exchange resins, and then recovered by freeze-drying, and characterized by high-performance liquid chromatography (HPLC) using an amino column and a mixed solvent of water and acetonitrile, as can be seen in Figure 2. As reference, this figure also contains an HPLC chart for standard samples, i.e., two-, three-, four-, five-, and six-carbon sugar alcohols (ethylene glycol, glycerol, erythritol, D-arabinitol, and D-mannitol, respectively) (Figure 2a). In Figure 2b–d, the signals at ca. 5 min may be due to impurities, e.g., inorganic salts. As can be seen in Figure 2b, the HPLC chart for the formose reaction in the absence of boronic acid compounds contains a number of signals in a wide range of elution time, indicative for the formation of a complicated mixture. On the other hand, the HPLC chart for SPB indicates a broad signal in the region of smaller carbon numbers (Figure 2c), and the chart for pVPB/NaSS exhibits signals in the region of larger carbon numbers (Figure 2d). These data are suggestive of modest selectivity of the formose reaction in the presence of boronic acid compounds.

**Figure 3:**
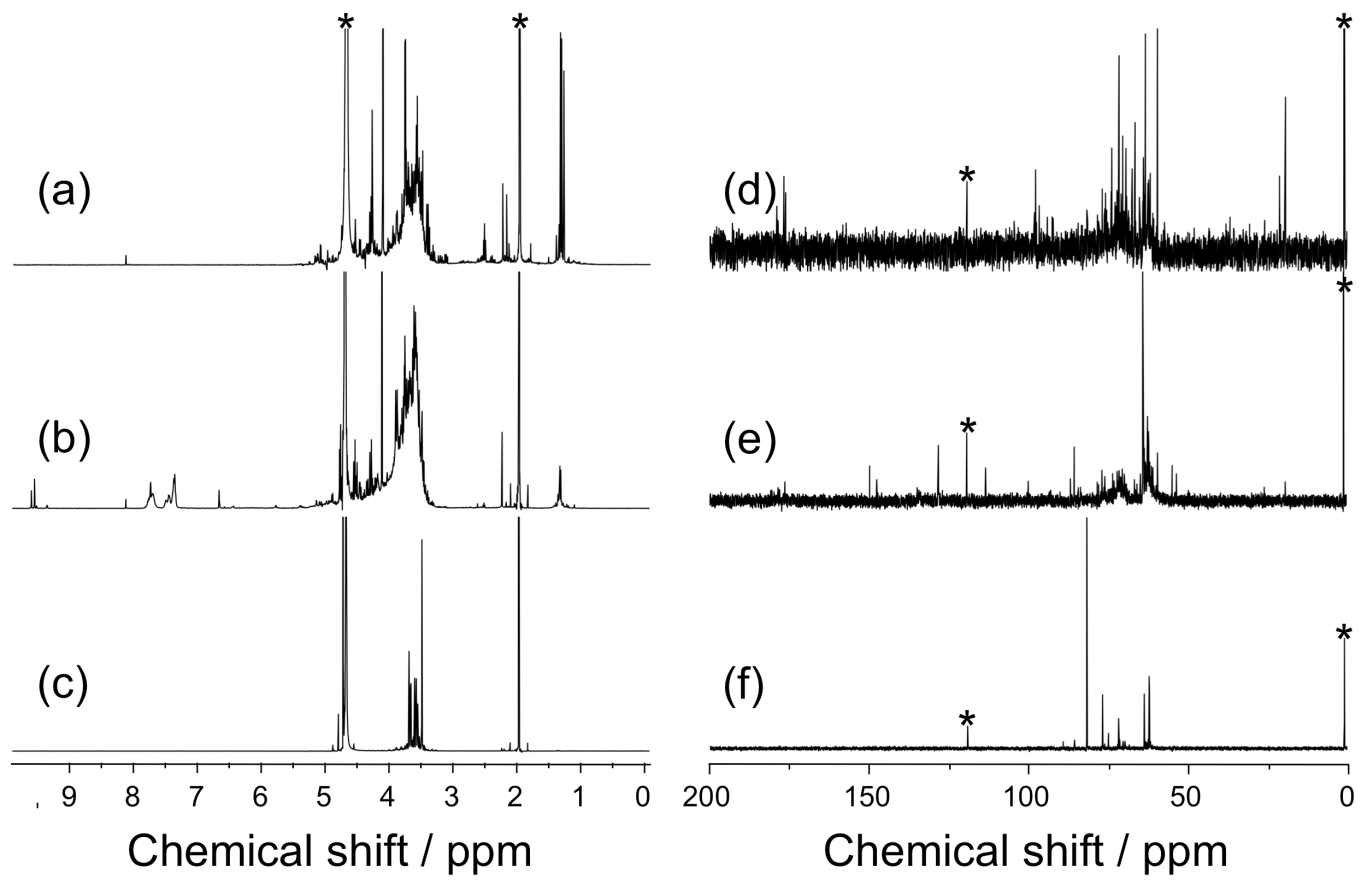
^1^H (a, b, and c) and ^13^C NMR spectra (d, e, and f) for the products of the formose reaction in the absence (a and d) and presence of 10 mM SPB (b and e) and 20 g L^−1^ pVPB/NaSS (c and f); the reaction times were 20, 90, and 180 min for (a and d), (b and e), and (c and f), respectively. Asterisks denote the signals of the internal standard, i.e., acetonitrile.

**Figure 4:**
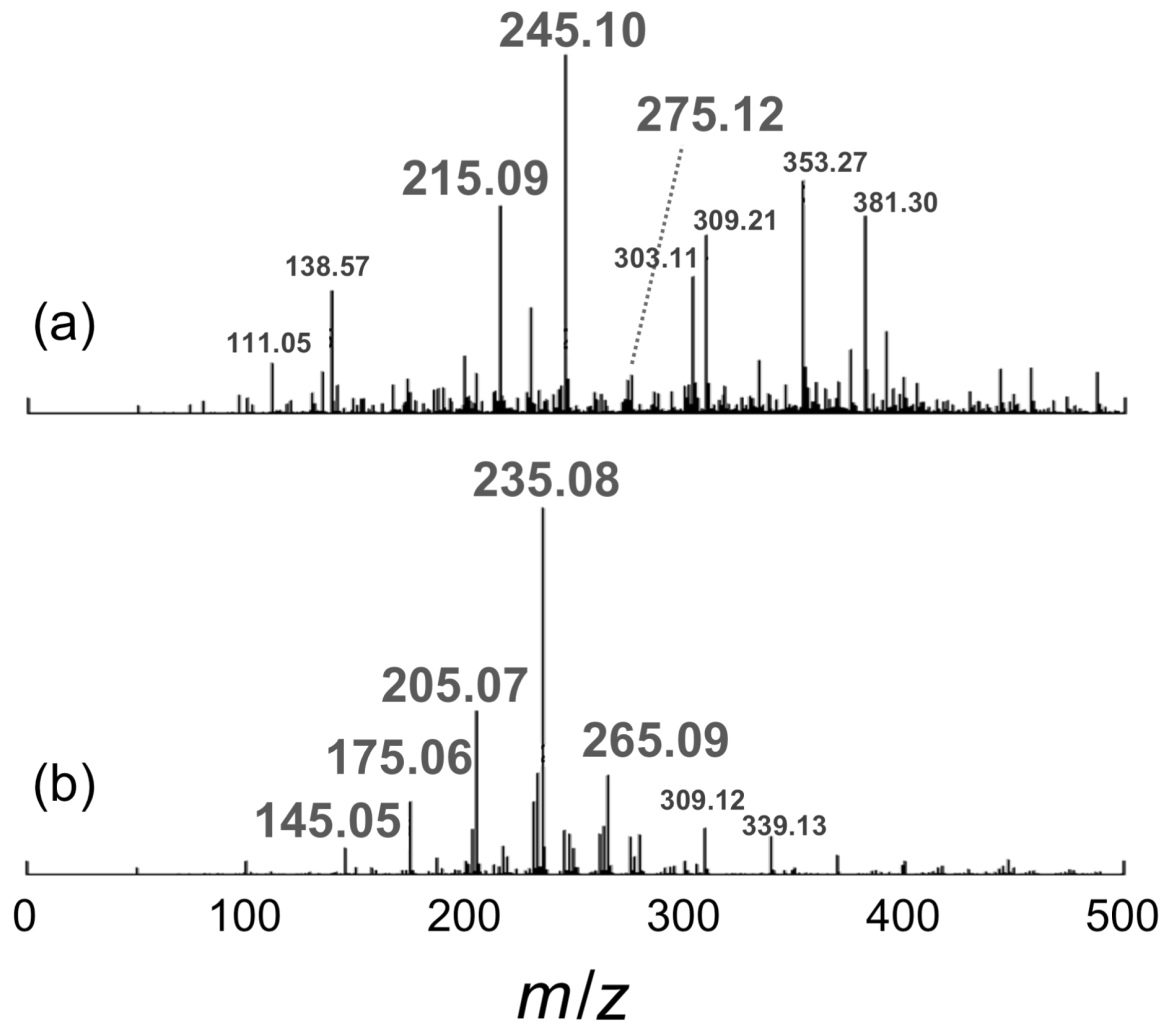
ESIMS data for the products of the formose reaction in the presence of 10 mM SPB (a) and 20 g L^−1^ pVPB/NaSS (b); the reaction times were 90 and 180 min for (a) and (b), respectively.

Figure 3 compares ^1^H and ^13^C NMR spectra for the products obtained in the presence of SPB and pVPB/NaSS. The ^1^H and ^13^C NMR spectra for SPB exhibit broad signals, similar to those for a formose reaction without boronic acid compounds. Since it was difficult to remove SPB from the reaction mixture, the NMR sample contained SPB, which might cause the broadening of NMR signals presumably because of equillibria of the formation of boronic acid esters. On the other hand, the ^1^H and ^13^C NMR spectra for pVPB/NaSS show well-resolved signals, indicating that the formose reaction provides products with a significant selectivity in the presence of pVPB/NaSS. Figure 4 displays typical examples of electrospray ionization mass spectrometry (ESIMS) data for the products of the formose reaction in the presence of SPB and pVPB/NaSS. The spectrum for SPB shows a series of signals at *m*/*z* = 215, 245, and 275, indicating an interval of 30 mass units, which corresponds to the formaldehyde unit. These signals are ascribable to potassium adducts of boronic acid esters formed from SPB and three-, four-, and five-carbon sugars. On the other hand, the spectrum for pVPB/NaSS contains a series of signals at *m*/*z* = 145, 175, 205, 235, and 265. These signals are assignable to sodium adducts of four to eight carbon sugar alcohols. On the basis of these characterization data, we can conclude that the formose reaction in the presence of SPB produces favorably sugars of a small carbon number whereas the formose reaction in the presence of pVPB/NaSS produces preferably sugar alcohols of a larger carbon number.

Here we discuss the effect of SPB and pVPB/NaSS on formose reaction. As shown in Figure 1, both SPB and pVPB/NaSS retarded the formose reaction, indicating that boronic acid compounds form esters with the sugars and sugar alcohols formed and the esters somehow protect the sugars or sugar alcohols from further reaction. The ESIMS data of products elucidated that the formose reactions in the presence of SPB and pVPB/NaSS exhibited different selectivities; sugars of a small carbon number were formed preferably in the presence of SPB, whereas sugar alcohols of a larger carbon number were obtained favorably in the presence of pVPB/NaSS (Figure 4). The selectivity of products should be dependent on the stability of esters. It is thus likely that SPB forms rather stable esters with sugars of a small carbon number and pVPB/NaSS forms relatively stable esters with sugar alcohols of a larger carbon number. The detailed mechanism of selective formose reaction assisted with boronic acid compounds should be investigated in the near future.

In order to control the formose reaction, we carried out formose reactions in the presence of SPB and pVPB/NaSS. The time–conversion data indicate that SPB and pVPB/NaSS retarded the formose reaction. The characterization data by HPLC, NMR, and ESIMS for the products indicated that sugars of a small carbon number were formed favorably in the presence of SPB, whereas sugar alcohols of a larger carbon number were formed preferably in the presence of pVPB/NaSS.

## Supporting Information

File 1 Experimental section and time–conversion plots for formose reactions in the presence of SPB and pVPB/NaSS.
