# Supplementary material for: Formose reaction controlled by boronic acid compounds
Source: Beilstein J Org Chem. 2016 Dec 8;12:2668–72. doi: 10.3762/bjoc.12.263 (PMC5238582; doi:10.3762/bjoc.12.263)
Supplement: File 1 — Experimental section and time–conversion plots for formose reactions in the presence of SPB and pVPB/NaSS. [file Beilstein_J_Org_Chem-12-2668-s001.pdf]

Supporting Information

for

Formose reaction controlled by boronic acid compounds

Toru Imai, Tomohiro Michitaka, and Akihito Hashidzume*

Address: Department of Macromolecular Science, Graduate School of Science, Osaka University, 1-1 Machikaneyama-cho, Toyonaka, Osaka 560-0043, Japan

Email: Akihito Hashidzume - hashidzume@chem.sci.osaka-u.ac.jp

*Corresponding author

Experimental section and time–conversion plots for formose reactions in the presence of SPB and pVPB/NaSS

Experimental Section

Materials

Phenylboronic acid and 4-vinylphenylboronic acid (VPB) were purchased from Tokyo Chemical Industry. An aqueous solution of formaldehyde (36 wt %), calcium hydroxide, and sodium 4-styrenesulfonate (NaSS) were purchased from Sigma-Aldrich Japan. 4,4′-Azobis(4-cyanovaleric acid) (ACVA) was purchased from Wako Pure Chemical Industries. Water was purified by a Millipore Milli-Q system. Other reagents were used without further purification.

Sodium phenylborate (SPB) was prepared by neutralization of phenylboronic acid with an equimolar amount of sodium hydroxide.

VPB (0.45 g, 3.0 mmol), NaSS (6.2 g, 30 mmol) and ACVA (0.12 g, 0.44 mmol) were dissolved in a mixed solvent of water (30 mL) and DMF (12 mL) under an argon atmosphere. The reaction mixture was warmed with an oil bath thermostated at 60 °C for 24 h. The polymerization was quenched by rapid cooling with an ice-water bath. An aqueous solution of sodium hydroxide (1 M, 20 mL) was added to the reaction mixture for neutralization. The neutralized polymer sample was purified by dialysis for a week.

Measurements

Size exclusion chromatography equipped with an online multi-angle light scattering detector (SEC-MALS) measurements were performed on a JASCO GPC-101 system equipped with a Wyatt DAWN HELEOS-II multiangle light scattering photometer and a Shodex SB-806M HQX2 column using 0.1 M NaCl as eluent at 30 °C with a flow rate of 1.0 mL min^{-1}.

UV–vis spectra for the acetylacetone method were recorded on a Jasco V-500 spectrophotometer.

HPLC measurements for the formose samples were carried out on a Jasco LC-2000 Plus system equipped with a Jasco PU-2080 pump and a Shodex 5NH2P-50 4D column. A mixed solvent of water and acetonitrile (1/3, v/v) was used as eluent at a flow rate of 0.6 mL min^{-1}. The sample signals were detected using a Jasco RI-2031 detector.

^{1}H and ^{13}C NMR spectra were measured on a JEOL JNM ECS400 or ECA500 spectrometer using $CDCl_3$ or D_2O as a solvent at 25 °C. Acetonitrile was used as an

internal standard. Chemical shifts in ^{13}C NMR were referenced to the signal due to the methyl carbon of acetonitrile (δ = 1.47 ppm).

ESIMS data were recorded in positive mode on a Thermo Fisher Scientific LTQ-Orbitrap-XL, controlled by the XCARIBUR 2.1 software package. Methanol was used as a solvent. The conditions of ionization were set to the following parameters; ion spray voltage at 3.5 kV, ion spray temperature at 100 °C, and ion transfer tube temperature at 275 °C. Internal calibration of ESIMS was carried out using the monoisotopic peaks of sodium adducted ion of diethylphthalate (*m*/*z* 314.1410), protonated ion of di-2-ethylhexylphthalate (*m*/*z* 391.2843), and sodium adducted ion of di-2-ethylhexylphthalate (*m*/*z* 413.2662).

Formose reaction for time–conversion experiments

Calcium hydroxide (23.7 mg, 0.32 mmol), a cocatalyst (glyceraldehyde or fructose, 0.11 mmol), and a boronic acid compound (SPB or pVPB/NaSS) were added to an aqueous solution of formaldehyde (200 mM, 16 mL). The reaction mixture was warmed with a water bath thermostated at 60 °C. After predetermined times, aliquots (0.1 mL) of the reaction mixture were taken. To the aliquots was added water (5 mL), and then the concentration of formaldehyde was determined by the acetylacetone method [s1,s2].

Preparation of the product for characterization

Calcium hydroxide (23.7 mg, 0.32 mmol), a cocatalyst (glyceraldehyde or fructose, 0.11 mmol), and a boronic acid compound (SPB or pVPB/NaSS) were added to an aqueous solution of formaldehyde (200 mM, 16 mL). The reaction mixture was warmed with a water bath thermostated at 60 °C. The reaction was then quenched with hydrochloric acid (1 M, 3 mL) at 0 °C using an ice bath. For the reactions using

the polymer samples, formose samples were purified by dialysis (1 L) overnight. Ion-exchange resins (Amberlite IR-120H and IRA-410) were added to the reaction mixture, and the mixture was stirred for 2–3 h. After filtration and evaporation, the product for characterization was recovered in ca. 20–30 % yield.

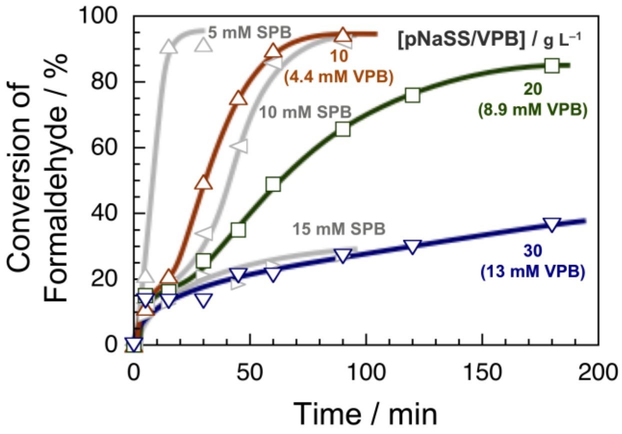

Figure S1: Time–conversion plots for formose reactions using 200 mM formaldehyde, 20 mM $Ca(OH)_2$, and 6.9 mM cocatalyst in the presence of SPB and pVPB/NaSS; fructose and glyceraldehyde was used as a cocatalyst for SPB and pVPB/NaSS, respectively. The curves are drawn as a guide for the eye.

References

s1. http://www.maff.go.jp/nval/kijyun/pdf/ST06130.PDF.

s2. Maruo, Y. Y.; Nakamura, J.; Uchiyama, M. *Talanta* **2008**, *74*, 1141-1147.
